# Zuo Jin Wan Reverses DDP Resistance in Gastric Cancer through ROCK/PTEN/PI3K Signaling Pathway

**DOI:** 10.1155/2018/4278568

**Published:** 2018-12-02

**Authors:** Meng-Yao Sun, Jian Sun, Jie Tao, Yu-Xia Yuan, Zhen-Hua Ni, Qing-Feng Tang

**Affiliations:** ^1^Department of Clinical Laboratory and Central Laboratory, Putuo Hospital, Shanghai University of Traditional Chinese Medicine, Shanghai 200062, China; ^2^Department of Clinical Laboratory, Shuguang Hospital, Shanghai University of Traditional Chinese Medicine, Shanghai 200021, China

## Abstract

Gastric cancer (GC) is the third leading cause of cancer-related death. Chemotherapy resistance remains the major reason for GC treatment failure and poor overall survival of patients. Our previous studies have proved that Zuo Jin Wan (ZJW), a traditional Chinese medicine (TCM) formula, could significantly enhance the sensitivity of cisplatin (DDP)-resistant gastric cancer cells to DDP by inducing apoptosis via mitochondrial translocation of cofilin-1. However, the underlying mechanism remains poorly understood. This study aimed to evaluate the effects of ROCK/PTEN/PI3K on ZJW-induced apoptosis in vitro and in vivo. We found that ZJW could significantly activate the ROCK/PTEN pathway, inhibit PI3K/Akt, and promote the apoptosis of SGC-7901/DDP cells. Inhibition of ROCK obviously attenuated ZJW-induced apoptosis as well as cofilin-1 mitochondrial translocation, while inhibition of PI3K had the opposite effects. In vivo, combination treatment of DDP and ZJW (2000 mg/kg) significantly reduced tumor growth compared with DDP alone. Moreover, the combined administration of ZJW and DDP increased the expression of cleaved ROCK and p-PTEN while it decreased p-PI3K and p-cofilin-1, which was consistent with our in vitro results. These findings indicated that ZJW could effectively inhibit DDP resistance in GC by regulating ROCK/PTEN/PI3K signaling and provide a promising treatment strategy for gastric cancer.

## 1. Introduction

GC is one of the most prevalent cancers characterized by high morbidity and mortality [1]. Surgery remains the only curative therapeutic methods so far. However, due to its late disease presentation, GC was detected in most of the patients at an advanced stage when the tumor is usually migrated, causing an extremely low 5-year survival rate [2]. For advanced gastric cancer, chemotherapy is the preferred therapeutic strategy. DDP is considered as a common drug for the treatment of GC [3]. DDP based adjuvant chemotherapy has been approved to increase survival after gastric resection [4]. Unfortunately, intrinsic or acquired drug resistance seriously limits the treatment effect of DDP [4]. Therefore, it is necessary to develop effective strategies to increase the sensitivity of DDP in GC treatment.

ZJW, a traditional Chinese medicine formula, composed of* Rhizoma Coptidis* (Huanglian in China) and* Evodia Rutaecarpa* (Wuzhuyu in China) with ratio of 6:1, has been used to treat gastrointestinal diseases in China for a long time and showed better therapeutic effects in adjuvant treatment of tumors [5–7]. Our previous studies have demonstrated that ZJW could significantly enhance the sensitivity of DDP-resistant gastric cancer cells to DDP and executed their biological effects by increasing mitochondrial apoptosis via PP1 and PP2A induced the dephosphorylation of p-cofilin-1, which implied that ZJW might serve as a synergistic drug with DDP in the treatment of gastric cancer [7]. However, it remains unclear how ZJW inhibits the DDP resistance in gastric cancer cells involving the modulation of cofilin-1 activity.

It is well known that the phosphatidylinositol 3-kinase/protein kinase B (PI3K/Akt) pathway plays important roles in mediating the multiple biological processes in tumor cells, including proliferation, apoptosis, and migration [8–11]. In addition, the PI3K/Akt pathway was demonstrated to function as a crucial pathway in the regulation of multidrug resistance (MDR) of cancer cells by modulating the activity of various MDR-related proteins, such as MDR1 and P-gp [12–14]. Recent evidence revealed that PI3K/Akt activation is strongly correlated with the inactivation of the tumor suppressor gene phosphatase and tensin homolog (PTEN) [15]. In tumor cells, at the upstream of Akt, PTEN serves as a phosphatase to block the activity of PI3K/Akt, promoting the cell proliferation, migration, and MDR [16–18]. Inactivation of PTEN is a crucial event in tumorigenesis and tumor development. Furthermore, PTEN has been identified as a new Rho-associated kinase (ROCK) substrate, and the activation of ROCK/PTEN appears to be involved in negative regulation of PI3K/Akt signaling [19, 20]. Our previous findings suggested that activation of PP1 and PP2A contributes to ZJW-induced mitochondrial apoptosis and translocation of cofilin-1 [7]. Notably, PP1 and PP2A are negatively regulated by PI3K/Akt pathway [21, 22]. Therefore, we speculated that the mechanism of ZJW-reversed resistance to DDP may be associated with the PI3K/Akt signaling pathway, which was mediated by ROCK1/PTEN, and finally induced PP1 and PP2A mediated dephosphorylation of p-cofilin-1 and mitochondrial translocation of cofilin-1.

In the present study,* in vitro* as well as* in vivo* studies were preformed to evaluate the role of ROCK/PTEN/PI3K in ZJW-reversed DDP resistance of gastric cancer. Our work may not only shed a light on the improvement of GC chemotherapy, but also provide evidence for further clinical investigation.

## 2. Materials and Methods

### 2.1. Cell Lines and Cultures

Human gastric cancer cell SGC7901 was provided by the Shanghai Cell Collection (Shanghai, China). Cells were cultured in RPMI-1640 medium (Gibco Laboratories, USA) containing 10% (v/v) fetal bovine serum, 1% penicillin–streptomycin 100 U/ml penicillin, and 100 *μ*g/ml streptomycin in a humidified atmosphere of 5% CO_2_ in air at 37°C.

DDP-resistant SGC7901/DDP cells were induced from SGC7901 cells, using a concentration gradient method to increase the half maximal inhibitory concentration (IC50) of DDP (as previously described) [7].

### 2.2. Preparation of the ZJW Extracts

Two herbs (*Rhizoma Coptidis* and* Fructus evodiae*) were from TCM pharmacy of Putuo Hospital, Shanghai University of Traditional Chinese Medicine (Shanghai, China). ZJW extracts were prepared as previously described [7].

### 2.3. Western Blot Analysis

Cell treated as indicated were harvested. The protein concentration was analyzed by BCA protein Assay Reagent (Sangon Biotech, Shanghai, China). Soluble lysates containing about 20 *μ*g proteins per sample were resolved with sodium dodecyl sulfate-polyacrylamide gel electrophoresis (SDS-PAGE) and then transferred onto polyvinylidene fluoride membranes. After blocking with 5% BSA, membranes were incubated with primary antibodies at 4°C overnight and secondary antibodies at room temperature for 1 h. The membrane signals were detected using an Enhanced Chemiluminescent Western Blotting Detection System (Millipore, Billerica, MA, USA) in accordance with the manufacturer's instruction. Antibodies against ROCK, PTEN, p-PI3K, PI3K, p-Akt, Akt, cofilin-1, p-cofilin-1, and GAPDH were from Cell Signaling Technology (Danvers, MA, USA). Antibodies against cleaved ROCK were purchased from Abcam (Cambridge, MA, USA). An anti-PP1 antibody was obtained from Santa Cruz Biotechnology (Dallas, TX, USA).

### 2.4. Cell Cycle and Apoptosis Assays

SGC-7901/DDP cells were treated as indicated. After 48 h of treatment, cells were collected and prepared to cell cycle and apoptosis assays. For cell apoptosis analysis, cells were stained with Annexin V–fluorescein isothiocyanate (FITC) apoptosis detection kit (BD Biosciences, San Jose, CA, USA) and proportions of apoptotic cells were also analyzed using flow cytometry (BD Biosciences). For cell cycle analysis, cells were fixed in ethanol at -20°C overnight; after washing with PBS three times, cells were stained with propidium iodide (PI, Sigma, St. Louis, MO, USA) at 37°C for 30 min. The cell cycle distribution was assessed with flow cytometry (BD Biosciences, Franklin Lakes, NJ, USA).

### 2.5. Immunofluorescent Staining

SGC-7901/DDP cells 5000 per well were seeded on cover slips precoated with 0.01% poly-lysine in a 24-well chamber. After drug treatments, cells were fixed with 4% paraformaldehyde for 20 min, permeabilized with 0.1% Triton X-100 for 10 min, and blocked with 5% bovine serum albumin (BSA) for 60 min at room temperature. Cells were probed with primary antibody at 4°C overnight and Alexa Fluor 488–conjugated goat anti-rabbit IgG (Life Technologies) in the dark for 1 h at room temperature. After washing, images were captured with a fluorescence microscope (Leica, Wetzlar, Germany).

### 2.6. In Vivo Studies

A total of 36 male athymic nude mice (4-to-6-week old) were randomly divided into six groups (6 mice/group): a negative control group, a DDP group (0.6 mg/kg), a ZJW group (2000 mg/kg), a DDP plus 2000 mg/kg ZJW group, a DDP plus 1000 mg/kg ZJW group, and a DDP plus 500/kg ZJW group; SGC-7901/DDP cells (1 × 10^6^ cells per mouse) were subcutaneously injected into the six groups of mice, respectively. The rats were orally administered ZJW and received intraperitoneal injection of DDP every 2 days for 4 weeks. The saline was used as control. Finally, the nude mice were killed and tumor tissues were excised and weighed.

### 2.7. Hematoxylin-Eosin (HE) Staining

Paraffin-embedded tissues were sliced into 4-*μ*m-thick sections and stained with hematoxylin and eosin.

### 2.8. Immunohistochemistry Staining

Tumor tissues were collected, fixed with 10% neutral-buffered formalin, dehydrated, paraffin-embedded, and sectioned by microtome. The sections were then incubated overnight at 4°C with primary anti-p-cofilin-1, anti-cleaved ROCK, and anti-p-PTEN antibodies. HRP-conjugated anti-secondary IgG (Cell Signaling Technology) was next applied for 30 min at room temperature. Color was developed for 3 min by incubation with 3,3′-diaminobenzidine (Sigma). Sections were counterstained with hematoxylin and examined under microscope.

### 2.9. Statistical Analysis

*In vivo *data are presented as the mean ± SEM and* in vitro* data are presented as the mean ± SD. Group means were compared using Student's t-test or one-way ANOVA followed by Dunnett's multiple-comparison test with GraphPad Prism version 5.01 (GraphPad Software, Inc., San Diego, CA, USA). P < 0.05 was considered as statistically significant.

## 3. Results

### 3.1. Modulation of ZJW on ROCK/PTEN/PI3K Signaling Pathway in SGC-7901/DDP Cells

**Figure 1:**
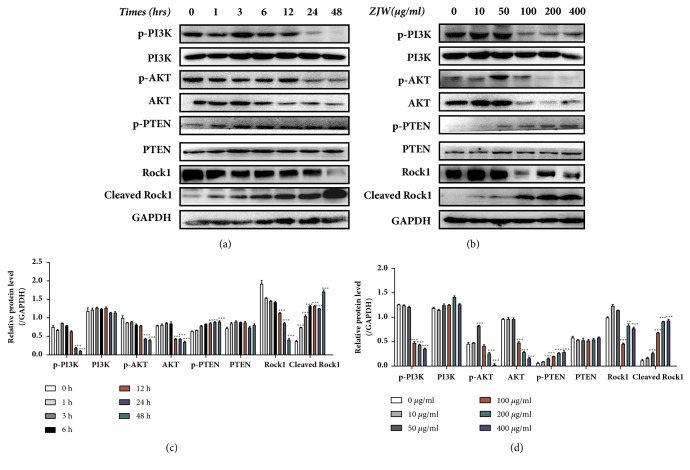
**ZJW effects on ROCK/PTEN/PI3K signaling pathway in SCG-7901/DDP**. (a) Cells were exposed to ZJW (50 *μ*g/mL) for 0-48 h. Western blotting was performed to evaluate protein levels involved in the ROCK/PTEN/PI3K pathway. (b) Cells were treated with the indicated concentration of ZJW for 48 h; representative images of three independent western blots (n = 3) are shown.

To investigate the effect of ZJW on ROCK/PTEN/PI3K pathway of gastric cancer, we examined the protein expression of ROCK/PTEN/PI3K in SGC-7901/DDP cells. SGC-7901/DDP cells were exposed to ZJW (50 *μ*g/mL) for 0, 1, 3, 6, 12, 24, and 48 h. We found that ZJW significantly increased activity of ROCK by assessing the expression level of cleaved ROCK. To determine whether PTEN is a downstream effector of ROCK in ZJW-regulated cell apoptosis, we detected the expression of PTEN and p-PTEN. The expression level of p-PTEN was upregulated in relation to control in a time dependent manner; however, the phosphorylation levels of PI3K and Akt were decreased in SGC-7901/DDP cells after ZJW 24 or 48 h treatment (Figure 1(a)).

Then SGC-7901/DDP cells were treated with different concentrations of ZJW (0, 10, 50, 100, 200, 400 *μ*g/mL) for 48 h (Figure 1(b)). In comparison with the control group (0 *μ*g/mL ZJW), ROCK, p-PI3K, Akt, and p-Akt expression were significantly reduced while cleaved ROCK and p-PTEN were significantly elevated in a dose-dependent manner. These results demonstrated that ZJW triggered ROCK/PTEN pathway and inhibited PI3K/Akt pathway in SGC-7901/DDP cells in a time- and dose-dependent manner.

### 3.2. ROCK, PI3K Inhibition Altered the Effect of ZJW in SGC-7901/DDP Cells

**Figure 2:**
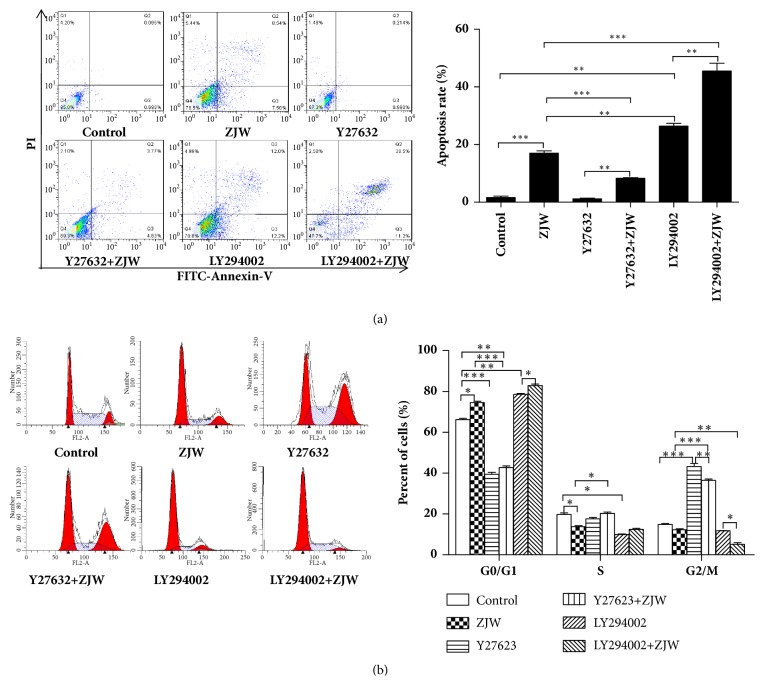
**Inhibition of ROCK and PI3K effects on the apoptosis and cycle induced by ZJW**. (a) Annexin V–FITC/PI double staining and flow cytometry were used to detect apoptosis in SCG-7901/DDP cells. (b) Cells were stained with PI and cell cycle distribution was analyzed by using flow cytometry. Experiments were performed three times independently. All data are represented as mean ± SD. ^*∗*^P < 0.05, ^*∗∗*^P < 0.01, ^*∗∗∗*^P < 0.001.

Given that ROCK and PI3K were major regulators of cell growth and apoptosis [19], ROCK inhibitor Y27623 or PI3K inhibitor LY294002 were used to assess the potential role of ROCK/PTEN/PI3K in ZJW increased chemosensitivity in SGC-7901/DDP cells. As shown in Figure 2(a), ZJW significantly promoted the apoptosis of SGC-7901/DDP cells. However, the combined exertion of ZJW and Y27623 yielded a significantly lower apoptosis rate than that of ZJW alone (Figure 2(a)). Furthermore, inhibition of PI3K obviously promoted ZJW-induced SGC-7901/DDP cells apoptosis. In addition, the notable increment of G2/M-phase cells was observed in SGC-7901 cells treated with ZJW and Y27623 as compared to ZJW alone (Figure 2(b)), when combined administration of ZJW and LY294002 inhibited the number of G2/M-phase phase cells. These data indicated that ROCK/PTEN/PI3K pathway contributed to the inhibitory effect of ZJW on drug resistance of SGC-7901/DDP cells.

### 3.3. Regulation of ROCK/PTEN/PI3K Signaling Pathway on ZJW-Mediated Dephosphorylation and Mitochondrial Translocation of Cofilin-1

**Figure 3:**
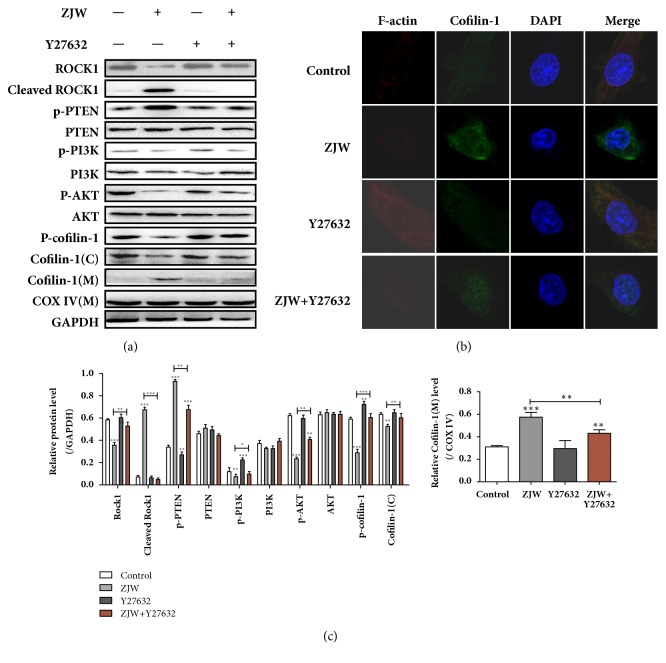
**ROCK1/PTEN/PI3K signaling pathway regulates ZJW-mediated mitochondrial translocation of cofilin-1**. Cells treated with ZJW (50 *μ*g/mL) were cotreated with or without Y27632 for 24 h, and western blotting was used to detect expression levels of proteins involved in the ROCK/PTEN/PI3K pathway (a). Depolymerisation of F-actin and translocation of cofilin-1 from the cytoplasm to the mitochondria in SGC7901/DDP cells were detected using an immunofluorescence assay (b). Quantification of protein levels (c). ^*∗∗*^P < 0.01, ^*∗∗∗*^P < 0.001 versus control.

We previously reported that changes in p-cofilin-1 and cofilin-1 protein expression levels were a potential mechanism of ZJW-mediated apoptosis in SGC-7901/DDP cells [7]. Therefore, we further validated whether the increased dephosphorylation and mitochondrial translocation of cofilin-1 in SGC-7901/DDP cells could be targeted by ROCK/PTEN/PI3K. We applied ZJW and ROCK inhibitor Y27623 separately and in combination, detecting the protein levels of ROCK, cleaved ROCK, p-PTEN, PTEN, p-PI3K, PI3K, Akt, p-Akt, p-cofilin-1, and cofilin-1. As shown in Figures 3(a) and 3(c), ZJW significantly activated ROCK/PTEN, inhibited PI3K/Akt, and induced mitochondrial translocation of cofilin-1 in SGC-7901/DDP cells, whereas Y27623 treatment had the opposite effect. In addition, the combination of ZJW and Y27623 reversed the ROCK/PTEN elevation, mitochondrial translocation of cofilin-1, and PI3K/Akt inhibition induced by ZJW.

Immunofluorescence images (Figure 3(b)) demonstrated that ZJW treatment induced the degradation of F-actin and aggregation of cofilin-1 in the mitochondria. Y27623-treated SGC7901/DDP cells exhibited an increased expression of F-actin, while SGC7901/DDP cells treated with ZJW and Y27623 showed a significantly lower F-actin degradation and cofilin-1 accumulation in mitochondria than that observed with ZJW alone. These results indicated that the activation of ROCK played an important role in ZJW-mediated dephosphorylation of p-cofilin-1.

### 3.4. PI3K Contributes to ZJW-Induced Activation of PP1/PP2A and Mitochondrial Translocation of Cofilin-1

**Figure 4:**
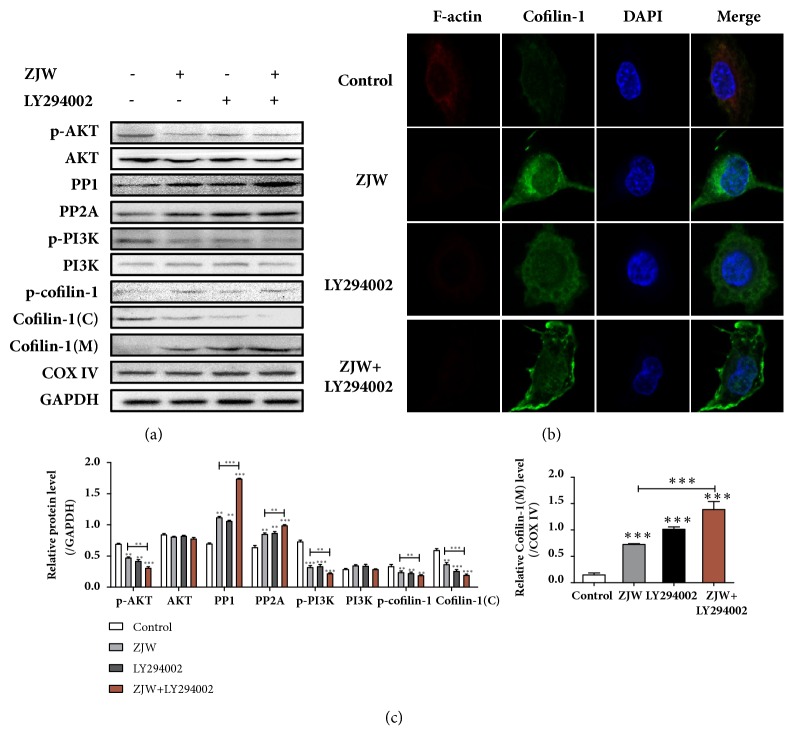
**PI3K contributes to ZJW-induced activation of PP1/PP2A and mitochondrial translocation of cofilin**. Cells treated with ZJW (50 *μ*g/mL) were cotreated with or without LY294002 for 24 h, and western blotting was used to detect expression levels of proteins involved in the PI3K/Akt pathway (a). Depolymerisation of F-actin and translocation of cofilin-1 from the cytoplasm to the mitochondria in SGC7901/DDP cells were detected using an immunofluorescence assay (b). Quantification of protein levels (c). ^*∗∗*^P < 0.01, ^*∗∗∗*^P < 0.001 versus control.

PI3K/Akt signaling pathway occupies a crucial position in regulating cell growth, viability, apoptosis, chemoresistance, etc. [23]. To further verify whether PI3K/Akt represented a key step in the ZJW-induced activation of PP1/PP2A and mitochondrial translocation of cofilin, the PI3K inhibitor LY294002 was performed. Western blotting analysis (Figures 4(a) and 4(c)) indicated that either ZJW or LY294002 alone could significantly repress the phosphorylation levels of PI3K and Akt and increase the activation of PP1/PP2A and mitochondrial translocation of cofilin-1. Moreover, cells cultured on both ZJW and LY294002 displayed decreased protein levels of p-PI3K, p-Akt, and cofilin-1(C), while the expressions of PP1, PP2A, and cofilin-1(M) were upregulated.

Immunofluorescence analysis (Figure 4(b)) demonstrated that ZJW or LY294002 treatment significantly increased the level of F-actin. However, there was no cofilin-1 accumulation observed. ZJW and Y27623 combined exerted further enhanced F-actin expression and cofilin-1 accumulation in mitochondria than that observed with ZJW alone. These results indicated that the activation of PI3K plays an important role in ZJW-induced activation of PP1/PP2A and mitochondrial translocation of cofilin-1.

### 3.5. ZJW Inhibits Tumor Growth in SCG-7901/DDP Xenografts Animal Model

**Figure 5:**
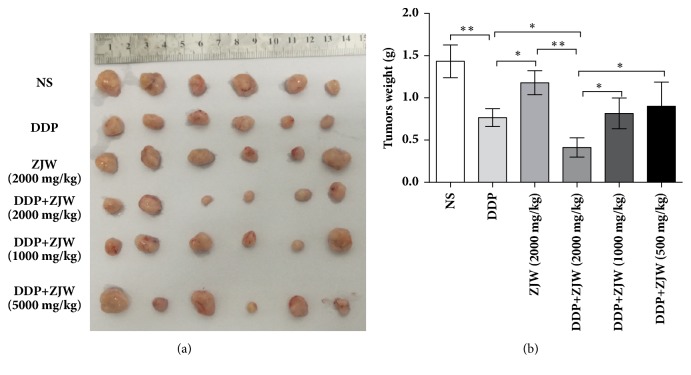
**ZJW inhibits tumor growth and induces apoptosis in SCG-7901/DDP xenografts animal model**. Xenograft mice were divided into six groups: negative control (NC), DDP, ZJW, DDP combined with 500 mg/kg ZJW, DDP combined with 1000 mg/kg ZJW, and DDP combined with 2000 mg/kg ZJW. After 30-time treatment the nude mice were killed and tumor tissues were excised and weighed. (a) Representative tumor tissues isolated from each treatment group. (b) The weight of the dissected tumors from each treatment group. Mean ± SE, n = 6. ^*∗*^P < 0.05, ^*∗∗*^P < 0.01.

Next, we determined whether the addition of ZJW increased the sensitivity of SCG-7901/DDP cells to DDP in vivo. Compared with control, DDP therapy exhibited significantly reduced tumor weights, but not ZJW. Mice receiving DDP and ZJW (2000 mg/kg) combination therapy exhibited significantly reduced tumor volumes compared with DDP alone (Figure 5). However, DDP and ZJW (1000 mg/kg) or DDP and ZJW (500 mg/kg) combination therapy did not show a significant alternation in tumor growth when compared to DDP group. It is possible that increasing the dose of ZJW may sensitize DDP-resistant cells to reach clinical efficacy. Taken together, with high dose of ZJW (2000 mg/kg), combination therapy could strongly suppress DDP-resistant gastric cancer xenograft tumor growth.

### 3.6. Effects of ZJW on ROCK/PTEN /PI3K and p-Cofilin-1 In Vivo

**Figure 6:**
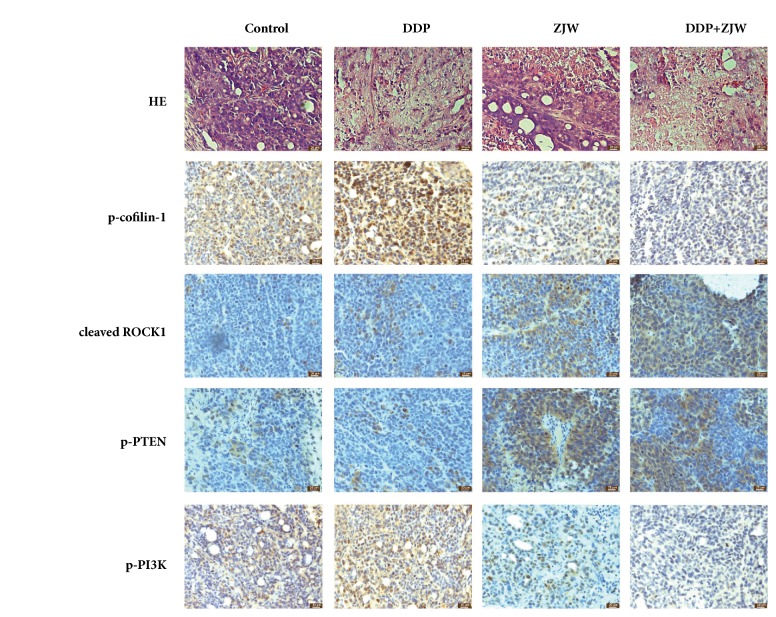
**The effects of ZJW on the expression ROCK/PTEN/PI3K signaling and p-cofilin-1 of tumor bodies in vivo**. (a) Representative HE staining of the indicated tumors of mice. (b) Representative photographs of IHC analysis of ROCK, PTEN, p-PI3K, and p-cofilin-1 in indicated tumors of mice.

The representative HE staining of the indicated tumors of mice was shown in Figure 6. Our results showed that tumors treated with the combination of DDP and ZJW (2000 mg/kg) showed more cell vacuolization and nuclear shrinkage than with DDP alone, which was closely associated with a decrease in the tumor size in the SCG-7901/DDP xenografts mice.

Furthermore, The expression of cleaved ROCK, p-PTEN, p-PI3K, and p-cofilin-1 was assessed using immunohistochemistry to evaluate the effect of ZJW in vivo. Similar to the in vitro results, the combination of ZJW and DDP increased the expression of cleaved ROCK and p-PTEN and decreased the expression of p-PI3K and p-cofilin-1 compared to DDP alone. These results suggested that ZJW increased the sensitivity of DDP in GC through ROCK/PTEN/PI3K and p-cofilin-1.

## 4. Discussion

DDP is a core component of chemotherapeutic treatment for gastric cancer. However, DDP resistance remains an obstacle to chemotherapy in tumor patients [24]. Our previous studies have shown that a combination of DDP with the herbal extraction ZJW could cause the mitochondrial apoptosis of DDP-resistant gastric cancer cells by dephosphorylation of p-cofilin-1 via activation of PP1 and PP2A [7], but the molecular mechanism remains largely unknown.

The ROCK/PTEN signaling pathway plays an important role in tumor cells apoptosis via mitochondrial translocation of cofilin-1. Activation of ROCK/PTEN could induce human prostate LNCaP cancer apoptosis by mitochondrial translocation of cofilin-1 [25]. It is clear that PI3K/Akt is essential for the development of resistance to carcinoma therapy [6] and PTEN is the main negative regulator of the PI3K/Akt pathway. Thus, the present study investigated whether the apoptosis in SCG-7901/DDP cells in response to ZJW was correlated with ROCK/PTEN/PI3K signaling pathway.

We found that ZJW had the ability to activate ROCK/PTEN signaling pathway in a time- and dose-dependent manner, and a high level of p-PTEN antagonizes the effects of PI3K/Akt in cells. More importantly, the suppression of ROCK dramatically attenuated ZJW-induced apoptosis, anti-proliferation, and p-cofilin-1 dephosphorylation while the inhibition of PI3K had the opposite effect, determining the regulatory effects of ZJW on SCG-7901/DDP associated interactions with ROCK/PTEN/PI3K signaling pathway.

Recent studies have suggested that various mechanisms contribute to PP1 and PP2A activity [26]. PI3K was identified as a negative regulator [21, 22]. In our study, ZJW was able to inhibit PI3K and Akt. Additionally, our results revealed that PI3K inhibitor not only increased ZJW-induced apoptosis, but also enhanced expression of PP1 and PP2A and translocation of cofilin-1, suggesting that ZJW might induce activation of PP1 and PP2A in DDP-resistant gastric cells by suppressing PI3K/Akt pathway. Furthermore, accumulating literature indicated that PTEN is an important negative regulator in PI3K/Akt signaling pathway, which plays a pivotal role in cell apoptosis, growth, and proliferation [16–18], while its expression and activity can be regulated by ROCK [19]. Previous reports indicated that Rho/ROCK enhanced PTEN activity and, in the contrary, inhibited Akt activation. In MDA-MB-231 cells, the activation of RhoA/ROCK/PTEN signaling could inhibit the phosphorylation of PI3K and Akt, leading to mitochondria-mediated apoptosis [27]. Our experiment results were consistent with these reports and suggested that ROCK/PTEN activation and PI3K/Akt inhibition contributed to the cofilin-1 mitochondrial translocation and apoptosis induced by ZJW via PP1 and PP2A activation.

Preclinical* in vivo* evaluation of ZJW for anticancer drug resistance activity has also been conducted in subcutaneous xenograft models. A simple practice guide for dose conversion between animals and human was used to calculate the dose of ZJW and DDP used in our animal model. Consistent with the* in vitro* results, DDP and ZJW (2000 mg/kg) combination therapy significantly reduced tumor volumes compared with DDP alone (Figure 5). Moreover, the combined ZJW and DDP treatment markedly enhanced activation of ROCK and PTEN and suppressed PI3K/Akt, PP1 and PP2A activation mediated dephosphorylation of p-cofilin-1, and translocation of cofilin-1 from the cytoplasm into the mitochondria.

Studies revealed that PI3K signaling pathway could be activated after DDP treatment by EGFR, which is one of the main causes of DDP treatment failure for PI3K activation and appears to be involved in several chemotherapy resistance mechanisms [28]. Therefore, PI3K/Akt inhibition seems to be a promising approach to reverse chemoresistance in cancer therapy via targeting and negatively regulating PI3K signaling [29]. However, Phase I and II clinical trials utilizing PI3K inhibitor have shown there was no significant upregulation in survival time of advanced cancer patients [30–32]. Compared to PI3K/Akt inhibitors with serious side effects, the combination therapy using novel agents that are nontoxic, efficacious such as traditional Chinese medicine, and conventional chemotherapeutic agent like DDP significantly increased sensitization of cancer cells and reduced the toxicity of drugs via systemic regulation of multiple targets including PI3K/Akt [33–37]. Some studies have suggested that ZJW can be used as an adjuvant to many cancer therapies, improving efficacy and/or reducing adverse effects via multiple-target action like PI3K, NF-KB, and P-gp [6, 38, 39]. Thus, although the data in this study demonstrated that the ROCK/PTEN/PI3K signaling pathway plays a critical role in ZJW-induced cofilin-1 mitochondrial translocation and apoptosis via PP1 and PP2A activation, further studies are needed for future clinical use. Gastric carcinogenesis is a multistep process;* Helicobacter pylori*, a spiral-shaped Gram-negative bacterium, has been recognized as the causative agent for gastric cancer which was also associated with drug resistance [40–43]; multiple pharmacological effects of ZJW and its chemical constituents need to be learned to analyze its plausible role as chemopreventive agent in relation to* Helicobacter pylori*.

Alkaloids were proved to be the main ingredients in the treatment of digestive tract diseases [44]. Prior pharmacokinetics/pharmacodynamic studies revealed that the absorption, elimination, and systemic exposure level of these alkaloids were mainly influenced by the proportion of Coptidis Rhizoma and Evodiae Fructus, the pharmacological effect on gastrointestinal motility, and the physicochemical property of these alkaloids [45]. ZJW compatibilities reduced both Parameter apparent permeability coefficient (Papp)_basolateral→apical_ and efflux rate values of three indole alkaloids, and increased efflux rate values of two quinolone alkaloids from Evodiae Fructus [46]. Microemulsion gel delivery system can accelerate the transdermal absorption rate of ZJW, compared with the hydrogel drug delivery system, while bioavailability has no significant difference [47]. However, administration of ZJW inhibited moderately CYP2D6-mediated metabolism of dextromethorphan in healthy volunteers. The inhibition of CYP2D6 by ZJW could result in clinically relevant effects, either beneficial or deleterious, depending on the nature of the CYP2D6 genotype [48]. These findings would be helpful for a better understanding of the activities and clinical applications of ZJW.

## 5. Conclusion

In summary, we found that ROCK/PTEN/PI3K plays an important role in ZJW-reversed chemotherapeutic resistance of gastric cancer as a critical regulator of p-cofilin-1 dephosphorylation and mitochondrial translocation of cofilin-1. Understanding the precise role of ZJW in gastric cancer chemotherapeutic resistance and in ROCK/PTEN/PI3K signaling pathways increases our knowledge of the biological basis of cancer development and may also facilitate the development of new therapeutic strategies against gastric cancer.

## Data Availability

All data generated or analyzed during this study are included in this published article. Additional datasets used and/or analyzed during the current study are available from the corresponding author on reasonable request.

## Abbreviations

GC: Gastric cancer
ZJW: Zuo Jin Wan
TCM: Traditional Chinese medicine
DDP: Cisplatin
PI3K/Akt: Phosphatidylinositol 3-kinase/protein kinase B
MDR: Multidrug Resistance
PTEN: Phosphatase and tensin homolog
ROCK: Rho-associated kinase
NC: Negative control.
